# Prevalence and outcome of VEXAS syndrome in unrelated hematopoietic cell transplantation for bone marrow failure

**DOI:** 10.1007/s10238-025-01832-7

**Published:** 2025-08-22

**Authors:** Yoshitaka Zaimoku, Tatsuya Imi, Tatsuya Hatada, Hiroki Mizumaki, Hiroki Mura, Hiroki Yoshino, Yui Kano, Miku Kobayashi, Eriko Morishita, Natsumi Fushida, Takashi Matsushita, Keishi Mizuguchi, Hiroko Ikeda, Yasuhito Nannya, Seishi Ogawa, Kazuyoshi Hosomichi, Noriko Doki, Yuta Katayama, Takashi Koike, Ken-ichi Matsuoka, Tetsuya Nishida, Yoshiyuki Takahashi, Keisuke Kataoka, Hideyuki Nakazawa, Yasunori Ueda, Takahiro Fukuda, Tatsuo Ichinohe, Fumihiko Ishimaru, Makoto Onizuka, Yoshiko Atsuta, Toshihiro Miyamoto

**Affiliations:** 1 Department of Hematology, Kanazawa University Hospital, 13-1 Takaramachi, Kanazawa, Ishikawa 920-8641 Japan; 2 Innovative Clinical Research Center, Kanazawa University, Kanazawa, Japan; 3 Department of Clinical Laboratory Science, School of Health Sciences, College of Medical, Pharmaceutical and Health Sciences, Kanazawa University, Kanazawa, Japan; 4 Division of Health Sciences, Department of Clinical Laboratory Science, Graduate School of Medical Science, Kanazawa University, Kanazawa, Japan; 5 Department of Dermatology, Kanazawa University Graduate School of Medical Sciences, Kanazawa, Japan; 6 Department of Diagnostic Pathology, Kanazawa University Hospital, Kanazawa, Japan; 7 Division of Hematopoietic Disease Control, The Institute of Medical Science, The University of Tokyo, Tokyo, Japan; 8 Department of Pathology and Tumor Biology, Kyoto University Graduate School of Medicine, Kyoto, Japan; 9 Institute for the Advanced Study of Human Biology (WPI-ASHBi), Kyoto University, Kyoto, Japan; 10 Faculty of Medicine, Kindai University, Osaka-Sayama, Japan; 11 Laboratory of Computational Genomics, School of Life Science, Tokyo University of Pharmacy and Life Sciences, Tokyo, Japan; 12 Hematology Division, Tokyo Metropolitan Cancer and Infectious Diseases Center, Komagome Hospital, Tokyo, Japan; 13 Department of Hematology, Hiroshima Red Cross Hospital and Atomic-Bomb Survivors Hospital, Hiroshima, Japan; 14 Department of Pediatrics, Tokai University School of Medicine, Isehara, Japan; 15 Department of Hematology and Oncology, Okayama University Hospital, Okayama, Japan; 16 Department of Hematology, Japanese Red Cross Aichi Medical Center Nagoya Daiichi Hospital, Nagoya, Japan; 17 Department of Pediatrics, Nagoya University Graduate School of Medicine, Nagoya, Japan; 18 Division of Hematology, Department of Medicine, Keio University School of Medicine, Tokyo, Japan; 19 Division of Molecular Oncology, National Cancer Center Research Institute, Tokyo, Japan; 20 Department of Hematology and Medical Oncology, Shinshu University School of Medicine, Matsumoto, Nagano Japan; 21 Department of Hematology/Oncology, Transfusion and Hemapheresis Center, Kurashiki Central Hospital, Kurashiki, Japan; 22 Department of Hematopoietic Stem Cell Transplantation, National Cancer Center Hospital, Tokyo, Japan; 23 Department of Hematology and Oncology, Research Institute for Radiation Biology and Medicine, Hiroshima University, Hiroshima, Japan; 24 Japanese Red Cross Blood Service Headquarters, Tokyo, Japan; 25 Department of Hematology/Oncology, Tokai University School of Medicine, Isehara, Japan; 26 Department of Registry Science for Transplant and Cellular Therapy, Aichi Medical University School of Medicine, Nagakute, Japan; 27 Japanese Data Center for Hematopoietic Cell Transplantation, Nagakute, Japan

**Keywords:** VEXAS syndrome, *UBA1* mutation, Allogeneic hematopoietic cell transplantation, Bone marrow failure, Myelodysplastic syndrome

## Abstract

VEXAS syndrome (vacuoles, E1 enzyme, X-linked, autoinflammatory, and somatic) is a recently identified clonal disorder caused by somatic *UBA1* mutations in hematopoietic stem cells, leading to bone marrow failure (BMF) and systemic inflammation. We screened 1771 patients with BMF who underwent unrelated hematopoietic cell transplantation in Japan between 1995 and 2020 using multitarget real-time PCR. The diagnoses included myelodysplastic syndrome (MDS, *n* = 1139), myeloproliferative neoplasms (*n* = 125), plasma cell neoplasms (*n* = 23), acquired BMF (*n* = 395), and congenital BMF (*n* = 89). Pathogenic *UBA1* mutations were detected in two male patients with MDS (aged 48 and 63 years), corresponding to a prevalence of 0.11% in the overall cohort and 0.18% in MDS cases; an additional 70-year-old male was diagnosed outside of the cohort. All three underwent unrelated bone marrow transplantation following fludarabine and busulfan-based conditioning. The first and third patients died of idiopathic pneumonia syndrome 5 and 28 months after transplantation. In the third patient, *UBA1*-mutant cells persisted at low frequency in skin graft-versus-host disease tissue despite clearance from his blood. The second patient survived without relapse or graft-versus-host disease at 28 months. Although VEXAS syndrome is rare among unrelated HCT recipients with malignant and non-malignant BMF in the historical cohort, HCT is positioned as a potentially curative, yet high-risk strategy. Additional studies are essential to refine patient selection, optimize transplant timing, and improve management strategies to mitigate risk and enhance survival. Therefore, the role of tissue-residual *UBA1*-mutant clones in post-transplant complications warrants further investigation.

**Supplementary Information:**

The online version contains supplementary material available at 10.1007/s10238-025-01832-7.

## Introduction

Vacuole, E1 enzyme, X-linked, autoinflammatory, and somatic (VEXAS) syndrome is a recently identified monogenic disorder caused by clonal expansion of hematopoietic stem cells with somatic mutations in the *UBA1* gene on the X chromosome.^1,2^

*UBA1* encodes the ubiquitin-activating enzyme E1, which initiates the ubiquitination cascade essential for directing misfolded, damaged, and other proteins for proteasomal degradation, thereby regulating protein turnover and maintaining cellular proteostasis*.*^3,4^ In VEXAS syndrome, pathogenic *UBA1* mutations mainly involve three missense mutations at methionine-41 (p.Met41Leu, p.Met41Val, and p.Met41Thr) that selectively impair the expression of the cytoplasmic isoform UBA1b while preserving the nuclear isoform UBA1a.^1,5^ The reduction in cytoplasmic ubiquitylation leads to the accumulation of misfolded proteins and disruption of proteostasis, which in turn enhances the intrinsic production of pro-inflammatory cytokines.^1,6^ The resulting inflammation not only contributes to disease manifestations but also suppresses the normal hematopoietic stem cell function and drives clonal expansion of the *UBA1*-mutant population.^2^

Clinically, VEXAS syndrome features bone marrow failure (BMF) with macrocytic anemia, systemic inflammatory symptoms (such as fever, skin rash, chondritis, pneumonia, vasculitis, and thrombosis), and distinctive cytoplasmic vacuolation in myeloid and erythroid precursors. It predominantly affects older men, as *UBA1* escapes X-chromosome inactivation.^7^ A subset of patients with VEXAS present with myelodysplastic syndrome (MDS) or plasma cell neoplasms, typically with low-risk features.^8,9^ Somatic mutations in non-*UBA1* genes can coexist with the canonical *UBA1* mutations, but they usually display a spectrum of age-related clonal hematopoiesis rather than direct disease pathology.^8–10^

The prevalence of pathogenic *UBA1* mutations, independent of selection bias for inflammatory symptoms, is approximately 0.02% (1 of 4269) in men and 0.004% (1 of 26,238) in women over 50 years of age.^11^ The prevalence rate among MDS patients is reported to be higher (0.5–1.3%).^10,12^ Data on benign BMF without dysplasia or inflammatory symptoms remain unexplored.

Systemic corticosteroids can initially alleviate symptoms in patients with VEXAS syndrome; however, relapse is common after tapering.^1^ Alternative treatments have been studied with limited experiences. Janus kinase pathway inhibitors^13–16^ and other cytokine-directed agents^16–18^ have demonstrated efficacy in controlling the inflammatory manifestations of VEXAS syndrome. However, these immunosuppressive therapies do not reduce the risk of hematologic disease progression or substantially lower the glucocorticoid requirements.^19^

Treatment strategies commonly used for hematologic malignancies may offer a more promising approach than conventional immunosuppressive approaches for VEXAS syndrome owing to its neoplastic nature. Notably, cytotoxic therapies such as azacitidine^13,20–28^ and intensive chemotherapy^29^ have been shown to reduce both the burden of VEXAS clones and inflammatory symptoms, although relapse remains common. Allogeneic hematopoietic cell transplantation (HCT) following cytotoxic conditioning is increasingly recognized as a potentially curative option, but it carries the risk of transplant-related morbidity and mortality.^30–36^

This study aimed to investigate the prevalence and outcomes of VEXAS syndrome among patients with benign and malignant BMF disorders who underwent unrelated HCT, using historical registry data and archived biological samples.

## Subjects and methods

### Participants

Patients with MDS, myeloproliferative neoplasms, plasma cell neoplasms, and acquired or congenitally benign BMF who underwent unrelated HCT between 1995 and 2020 in Japan were enrolled. Pre-transplant blood DNA and clinical data were sourced from the Japanese Data Center for Hematopoietic Cell Transplantation (JDCHCT).^37^ Informed consent for future genetic analyses was obtained from all participants at the time of their registration in the transplant registry. The present study protocol, including the opt-out option, was publicly disclosed on the JDCHCT website in accordance with the Declaration of Helsinki. Ethical approval was granted by the Human Genome/Gene Analysis Research Ethics Committee of Kanazawa University.

### *UBA1* mutation detection

A multitarget real-time PCR assay was developed to simultaneously detect three pathogenic *UBA1* mutations, p.Met41Leu (c.121A > C), p.Met41Val (c.121A > G), and p.Met41Thr (c.122 T > C), along with the wild-type *UBA1*. The assay design included a primer pair, three variant-allele-specific locked nucleic acid (LNA) probes labeled with fluorescein, an LNA probe specific to the wild-type *UBA1* allele labeled with SUN fluorochrome, and a peptide nucleic acid (PNA) clamping probe complementary to wild-type *UBA1*. The PNA probe was incorporated to suppress wild-type *UBA1* amplification, while enhancing the detection of variant alleles. An analysis was conducted using a QuantStudio Pro 6 real-time PCR system (Thermo Fisher Scientific, Waltham, MA, USA).

Variant allele fractions (VAFs) were quantified using the QX200 droplet digital PCR system (Bio-Rad, Hercules, CA, USA) or the QuantStudio Absolute Q digital PCR system (Thermo Fisher Scientific) with the same primers and LNA probes employed in the real-time PCR assay. Mutations were confirmed by Sanger sequencing with PNA clamping, as previously described,^38^ using a distinct primer pair and a PNA probe. Sensitivity was validated by serial dilution of the synthesized mutant controls with genomic DNA from a healthy male donor.

Primers and LNA probes were synthesized by Integrated DNA Technologies (Coralville, IA, USA), whereas PNA probes were synthesized by Biologica (Nagoya, Japan). Targeted capture sequencing was performed in one patient to identify any somatic mutations associated with myeloid neoplasms, as previously described.^39,40^

## Results

### Participants

Among 4340 patients who underwent their first unrelated HCT in Japan between 1995 and 2020, pre-transplant samples were available for 1771 individuals and analyzed for *UBA1* mutations. The median age was 46 years (range, 0–75 years), and 62% of the patients were males. This male predominance reflects the overall demographics of the unrelated HCT registry, as 1586 (62%) of the 2569 patients without *UBA1* testing were males, consistent with the known male predominance in MDS.^41^

The diagnoses included MDS (*n* = 1139), myeloproliferative neoplasms (*n* = 125), plasma cell neoplasms (*n* = 23), acquired BMF (*n* = 395), and congenital BMF (*n* = 89). The distributions of the disease type, age, and sex across these diagnoses are summarized in Table 1.

**Table 1:** Diagnosis, age, and sex of patients

Parameter	Overall patients (*n* = 1771)	MDS (*n* = 1139)	MPN (*n* = 125)	Plasma cell neoplasm (*n* = 23)	Acquired BMF (*n* = 395)	Congenital BMF (*n* = 89)
***Classification, N (%)***		MDS-IB2, 345 (30%) MDS-IB1, 262 (23%) MDS-LB, 407 (36%) MDS-LB-RS, 32 (3%) MDS/MPN, 37 (3%) 5q^–^ syndrome, 5 (0.4%) RAEB-t, 25 (2%) RAEB, 20 (2%) Missing, 6 (0.5%)	CMML, 81 (65%) JMML, 42 (34%) PMF, 1 (0.8%) aCML, 1 (0.8%)	MM, 16 (70%) PCL, 5 (22%) Others, 2 (9%)	IAA, 346 (88%) HAAA, 22 (6%) PRCA, 10 (3%) PNH, 7 (2%) Others, 11 (3%)	FA, 33 (37%) CN, 20 (22%) DBA, 16 (18%) DC, 7 (8%) AMT, 4 (4%) Others, 9 (10%)
*Age at HCT (IQR), years*	46 (20–75)	54 (41–62)	44 (2–61)	54 (44–57)	19 (12–34)	6 (3–10)
Range	0–75	1–75	0–71	31–70	1–72	0–32
*Sex, N (%)*						
Male	1096 (62%)	738 (65%)	87 (70%)	15 (65%)	217 (55%)	39 (44%)
Female Missing	674 (38%) 1 (0.06%)	400 (35%) 1 (0.09%)	38 (30%)	8 (35%)	178 (45%)	50 (56%)
*Patients aged* > *45 years, N (%)*						
Male	604 (34%)	525 (46%)	41 (33%)	11 (48%)	27 (7%)	0 (0%)
Female	304 (17%)	247 (22%)	20 (16%)	6 (26%)	31 (8%)	0 (0%)
*Patients aged* ≤ *45 years, N (%)*						
Male	492 (28%)	213 (19%)	46 (37%)	4 (17%)	190 (48%)	39 (44%)
Female	371 (21%)	153 (14%)	18 (14%)	2 (9%)	147 (37%)	50 (56%)
Missing	1 (0.06%)	1 (0.09%)				

*aCML* atypical chronic myelogenous leukemia, *AMT* amegakaryocytic thrombocytopenia, *CMML* chronic myelomonocytic leukemia, *CN* Congenital neutropenia, *DBA* Diamond-Blackfan anemia, *DC* dyskeratosis congenita, *FA* Fanconi anemia, *HAAA* hepatitis-associated aplastic anemia, *IAA* idiopathic aplastic anemia, *IB* increased blasts, *LB* low blasts, *JMML* juvenile myelomonocytic leukemia, *MM* multiple myeloma, *MPN* myeloproliferative neoplasm, *PCL* plasma cell leukemia, *PNH* paroxysmal nocturnal hemoglobinuria, *PRCA* pure red cell aplasia, *RAEB* refractory anemia with excess blast, *RAEB*-*t* refractory anemia with excess blast in transformation, *RS* ringed sideroblast

### *UBA1* mutations

A serial dilution analysis established the detection limits for multi-target real-time PCR as follows: 0.13% for p.Met41Leu (c.121A > C), 0.5% for p.Met41Val (c.121A > G), and 0.5–1.0% for p.Met41Thr (c.122 T > C) (Supplementary Fig. 1).

**Fig. 1:**
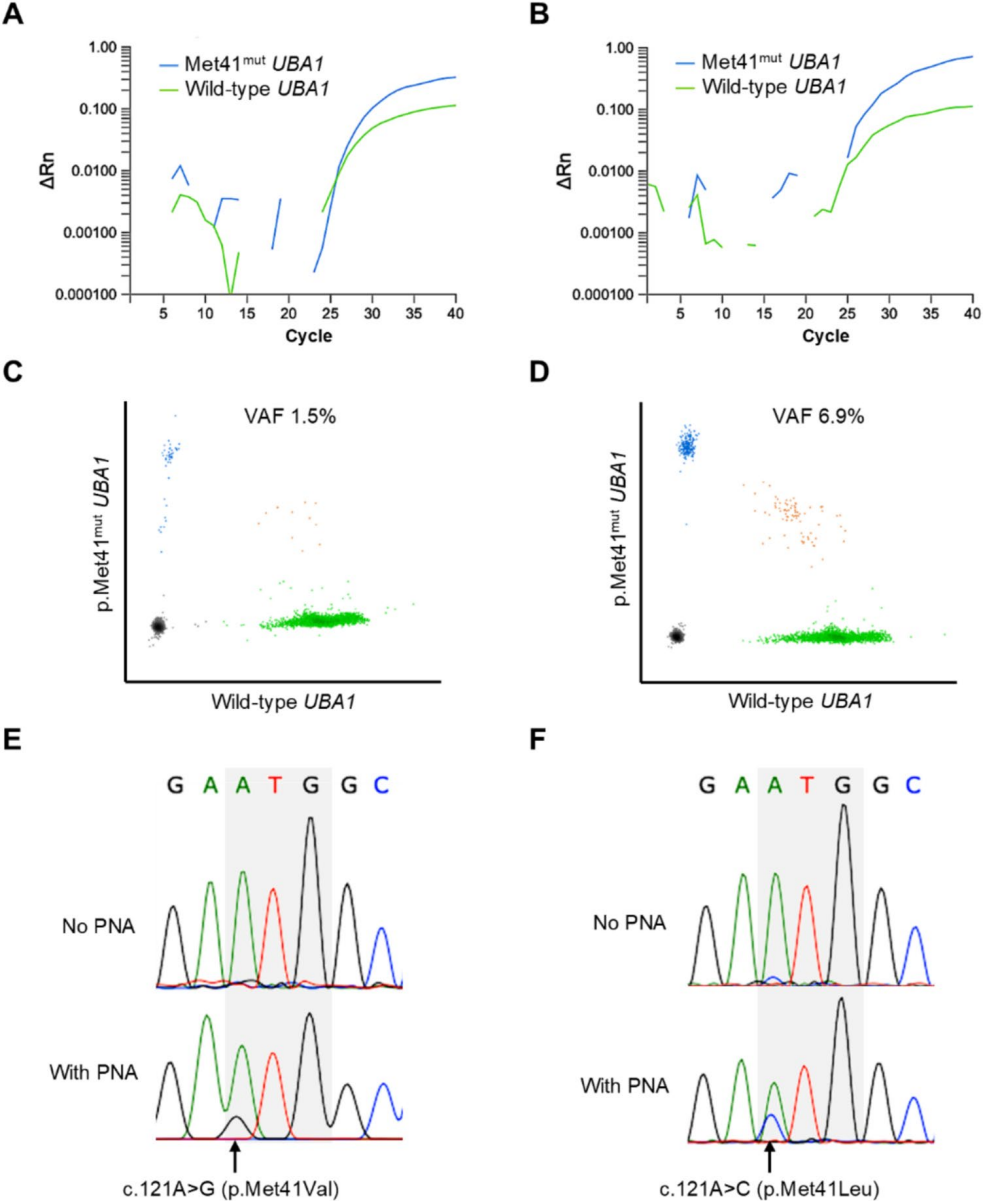
*UBA1* mutations in Cases 1 and 2. **a, b** *UBA1* mutations detected by multitarget real-time PCR with PNA clamping. **c, d** Digital PCR results: variant *UBA1* droplets (blue), wild-type *UBA1* droplets (green), droplets containing both alleles (orange), and droplets without any *UBA1* alleles (gray). **e, f** Sanger sequencing results with and without PNA clamping. The codon for methionine (ATG) at position 41 is highlighted in gray; arrows indicate the mutation site. panels **a, c**, and** e** correspond to case 1; panels **b, d**, and** f** to Case 2

In the cohort study, pathogenic *UBA1* mutations were identified in two of the 1771 patients (0.11%): p.Met41Val (VAF 1.5%) and p.Met41Leu (VAF 6.9%) (Fig. 1).

These low-frequency mutations were undetectable by conventional Sanger sequencing but were subsequently confirmed using PNA clamping. Both patients were male MDS patients of 48 and 63 years of age (Cases 1 and 2, Table 2). Additionally, we report another case of VEXAS syndrome with a p.Met41Thr mutation in a patient who underwent unrelated BMT at our hospital in 2022, outside the study period (Case 3).

**Table 2:** Three cases of VEXAS syndrome

Characteristic	Case 1	Case 2	Case 3
*UBA1* mutation (VAF)	p.Met41Val (1.5%)	p.Met41Leu (6.9%)	p.Met41Thr (35%)
Age at the diagnosis of MDS	48	63	70
Age at HCT	49	66	71
Sex	Male	Male	Male
Diagnosis	MDS-IB1	MDS-LB	MDS-LB
Cytogenetic abnormality	+ 8	Normal	+ 8 [15/20]
IPSS	Intermediate-2	Intermediate-1	Intermediate-1
Inflammatory symptoms	N/A	N/A	Fever, erythema nodosum
HCT-CI score	4 (arrhythmia, cardiovascular dysfunction, and lung function)	0	2 (cardiovascular dysfunction, cerebrovascular dysfunction,
Treatment before HCT	Induction chemotherapy, complete response	Azacitidine, partial response	Corticosteroid, partial response
Time from the diagnosis of MDS to HCT	15 months	29 months	8 months
HCT type	Unrelated BMT	Unrelated BMT	Unrelated BMT
Donor sex	Female	Male	Male
ABO disparity	Minor mismatch	Major mismatch	Match
HLA disparity (A, B, C, DRB1)	1/8 mismatch	Match	Match
Conditioning	FLU/BU2	FLU/BU4	FLU/BU2/TBI
GVHD prophylaxis	TAC + MTX	CSA + MTX	TAC + MTX + rATG
Engraftment	Day 16	Day 15	Day 19
Acute GVHD	Grade I (skin stage 2), spontaneous regression	None	Grade II (skin stage 3), regressed with systemic corticosteroid
Chronic GVHD	None	None	None
Chimerism after engraftment	Complete donor chimerism	Complete donor chimerism	Donor dominant > 95%
Complications	VZV reactivation	None	EBV reactivation, CMV retinitis, PCP
Outcome	Died of idiopathic pneumonia syndrome 5 months after BMT	Alive without relapse 28 months after BMT	Died of idiopathic pneumonia syndrome 28 months after BMT

*BU* busulfan, *CI* comorbidity index, *CMV* cytomegalovirus, *CSA* cyclosporin, *EBV* Epstein–Barr virus, *FLU* fludarabine, *IPSS* international prognostic scoring system, *PCP* pneumocystis pneumonia, *rATG* rabbit antithymocyte globulin, *TAC* tacrolimus, *TBI* total body irradiation, *VZV* varicella zoster virus

Based on the 2 registry cases, the prevalence of pathogenic *UBA1* mutations was 0.18% among MDS patients (2 of 1139) and 0.38% among male MDS patients over 45 years of age (2 of 525).

### Case 1

A 48-year-old man with MDS (increased blasts-1, trisomy 8) received induction chemotherapy and achieved complete remission. The patient underwent BMT from an unrelated female donor who was mismatched at one of the eight HLA loci. Reduced-intensity conditioning with fludarabine and busulfan was administered because of preexisting arrhythmia and cardiac/pulmonary dysfunction. Engraftment with complete donor chimerism was achieved. Post-transplant complications included stage 2 acute skin GVHD, which was resolved without systemic therapy, and varicella zoster virus reactivation, which was treated effectively with acyclovir. However, the patient died five months after BMT due to idiopathic pneumonia syndrome.

### Case 2

A 63-year-old man with MDS (low blasts, normal cytogenetics) achieved a partial hematologic response to azacitidine therapy. Although he developed an invasive fungal infection, he successfully recovered and underwent BMT from an HLA-matched unrelated male donor 29 months after the diagnosis, following myeloablative conditioning with fludarabine and busulfan. Engraftment with complete donor chimerism was also confirmed. The patient remains alive without relapse, GVHD, or major complications at 28 months after BMT.

### Case 3

**Fig. 2:**
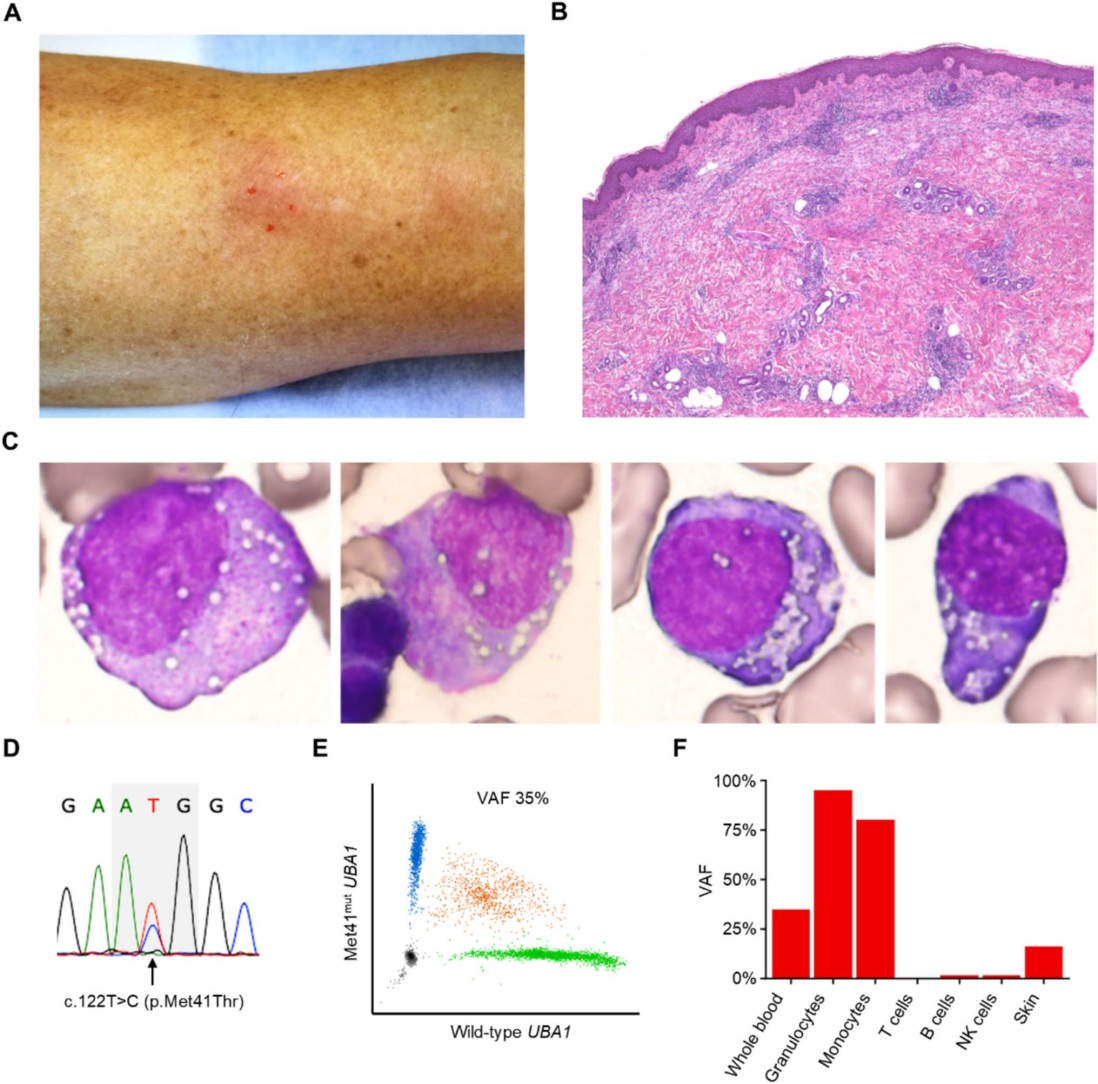
Clinical presentations and *UBA1* mutation analyses in Case 3. **a** A picture of the leg at the initial presentation. **b** A low-magnification image of the hematoxylin and eosin-stained section of the skin biopsy, showing perivascular lymphocytic infiltration in the superficial dermis. **c** Cytoplasmic vacuolization in bone marrow myeloid progenitors (first and second images) and erythroid progenitors (third and fourth images). **d** Sanger sequencing of *UBA1*. **e** Digital PCR results using whole blood DNA before BMT. **f** VAFs of the *UBA1* p.Met41Thr mutation detected in leukocyte subsets and a skin biopsy specimen at the diagnosis

A 70-year-old man was referred to our hospital with a one-year history of recurrent fever, erythema nodosum of the extremities (Fig. 2a), and macrocytic anemia that initially responded to systemic corticosteroid therapy but recurred upon tapering. The patient subsequently became transfusion-dependent for red blood cells and developed progressive thrombocytopenia (platelet count < 50 × 10^9^/L). Histological examination of the skin biopsy showed lymphocyte-predominant perivascular inflammation in the superficial dermis (Fig. 2b, Supplementary Fig. 2a–c). Bone marrow evaluation revealed dysplastic changes with significant vacuolations in myeloid and erythroid precursors (Fig. 2c), a blast count of 2.5%, and trisomy 8 in 15 of 20 metaphases, consistent with a diagnosis of MDS with low blasts.

*UBA1* analyses identified a p.Met41Thr mutation with a VAF of 35% in the peripheral blood (Fig. 2d, e). This mutation was predominantly present in myeloid cells (granulocytes and monocytes) but was almost absent in lymphocyte subsets (T cells, B cells, and NK cells; Fig. 2f, Supplementary Fig. 3a–f), consistent with previous reports.^1^ A further genomic analysis revealed no myeloid neoplasm-related somatic gene mutations. The same *UBA1* mutation was also detected in the skin biopsy specimen, with a VAF of 16% (Supplementary Fig. 3g).

Oral prednisolone at 10 mg/day was required to control the fever and skin lesions but had no hematologic effects. The patient remained transfusion-dependent, requiring red blood cell transfusions approximately every one to two weeks. Eight months after the diagnosis, the patient underwent BMT from an HLA-matched unrelated male donor following reduced-intensity conditioning with fludarabine, busulfan, and total body irradiation, with continued prednisolone administration (Supplementary Fig. 4). No significant early toxicities occurred after HCT, except for a neutropenic fever due to *Corynebacterium jeikeium* bacteremia on day 14, which resolved prior to neutrophil engraftment on day 19.

**Fig. 3:**
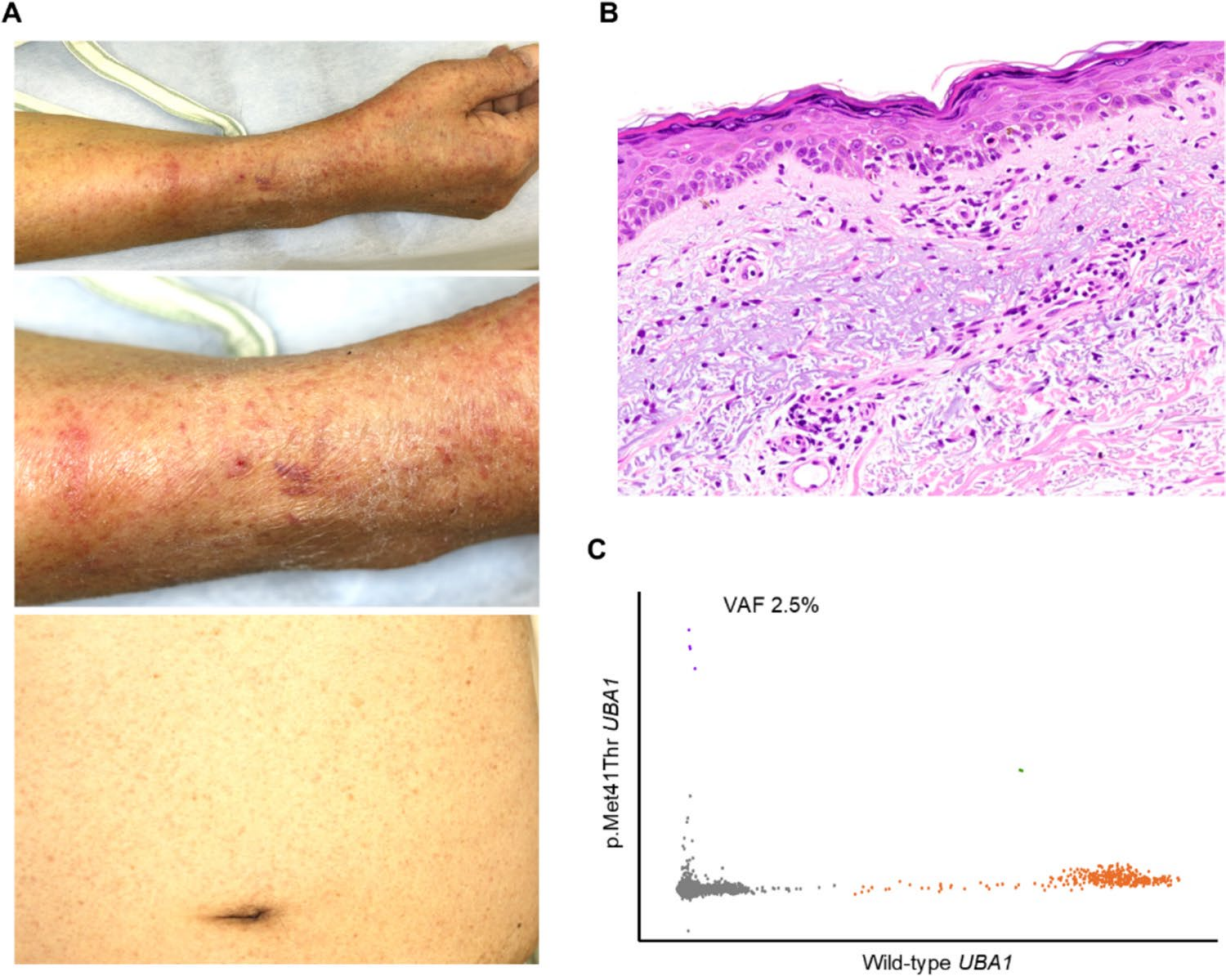
A *UBA1* analysis in the skin acute GVHD lesion from Case 3. **a** Pictures of acute skin GVHD involving the hand, upper arm, and abdomen. **b** Hematoxylin and eosin staining of the skin biopsy from the right hand reveals vacuolar degeneration at the dermoepidermal junction, with sparse intraepidermal lymphocytes, scattered necrotic keratinocytes, and perivascular lymphocytic infiltration in the superficial dermis, which are all consistent with a diagnosis of GVHD. **c** Digital PCR analysis of the skin GVHD biopsy specimen showing persistence of *UBA1*-mutant cells: variant *UBA1* droplets (purple: four dots), wild-type *UBA1* droplets (orange), droplets containing both alleles (green, two dots), and droplets without any *UBA1* alleles (gray)

On day 48, the patient developed grade II acute GVHD with stage 3 skin involvement (Fig. 3a, b, Supplementary Fig. 2d), which responded well to an increased prednisolone dose (20 mg/day). During the steroid tapering process, the patient developed pneumocystis pneumonia, Epstein–Barr virus reactivation without rituximab therapy, cytomegalovirus reactivation causing retinitis, and recurrent episodes of pneumonia, which were managed with antibacterial therapy. He ultimately died from respiratory failure secondary to idiopathic pneumonia syndrome 28 months after BMT.

A post-transplant digital PCR analysis of his blood obtained on day 20 and in subsequent assessments up to day 271 demonstrated the complete clearance of *UBA1* variant clones (Supplementary Fig. 5). In contrast, the skin biopsy specimen from acute GVHD obtained 48 days after BMT suggested a reduced, but persistent presence of *UBA1*-mutant cells, with a VAF of 2.5% (Fig. 3c).

## Discussion

Our study is the first to systematically evaluate the prevalence of pathogenic *UBA1* mutations across a broad range of acquired and inherited BMF disorders in a large nationwide unrelated HCT cohort. We identified pathogenic *UBA1* mutations in two patients with MDS from the registry cohort as well as a third case diagnosed outside the cohort, all of whom underwent unrelated BMT.

Despite employing a sensitive detection method, the observed prevalence of 0.2% among patients with MDS in our transplant registry was slightly lower than the previously reported rate of 0.5–1.3% but remained significantly higher than that observed in the general population.^10–12^ The low prevalence of VEXAS syndrome in our cohort likely reflects its typical clinical presentation as low-risk MDS in elderly patients who are generally less likely to receive unrelated HCT. Moreover, prior chemotherapy may selectively eliminate *UBA1*-mutant clones,^20–23,26,27,29^ thereby reducing the detectability of mutations. Indeed, the two cases identified after chemotherapy exhibited low VAFs, which were undetectable by Sanger sequencing.

In addition, we demonstrated that VEXAS syndrome is absent in benign BMF. This finding aligns with previous observations that pathogenic *UBA1* mutations are associated with distinct clinical features and characteristic bone marrow morphologic changes,^1^ minimizing the likelihood of misdiagnosis as non-malignant BMF.

Based on our findings, routine screening for *UBA1* mutations may be unnecessary in unrelated HCT recipients. However, testing should be performed with a low threshold in patients with relevant clinical features, such as macrocytic anemia, skin rash, or bone marrow vacuolations, as various targeted therapeutic options are emerging. Furthermore, *UBA1* mutation screening is simple, rapid, and minimally invasive, as demonstrated in this study.

The skin is the most commonly affected organ in VEXAS syndrome, characterized by leukocytoclastic vasculitis, neutrophilic dermatosis, or perivascular dermatitis,^1,42^ and is also a primary target of GVHD. We hypothesized that residual *UBA1*-mutant cells in the skin, such as macrophages, contribute to localized inflammation that mimics or triggers GVHD and other transplant complications. Indeed, we confirmed the persistence of these clones in the skin GVHD tissue specimens. These findings not only suggest that targeted therapies against *UBA1*-mutant clones, such as Janus kinase pathway inhibitors or hypomethylating agents, could offer clinical benefit in managing post-transplant complications but also raise the possibility that idiopathic pneumonia syndromes, the causes of death in two cases, may be attributed to persistent *UBA1*-mutant clones in the lung tissue, although autopsies were not performed in these patients.

At the time of clinical decision making for Case 3, no treatment had demonstrated proven efficacy for VEXAS syndrome, except for several case reports of successful HCT. Because the patient had severe anemia and thrombocytopenia and an HLA-matched unrelated donor was available, HCT was selected as the most appropriate treatment option.

Experience with HCT for VEXAS syndrome has largely been limited to small case series,^43,44^ and prospective trials are currently underway.^45^ Infection is the primary cause of post-HCT mortality, likely due to immunosuppression resulting from prior corticosteroid therapy and lymphopenia or dysfunctional immune responses associated with VEXAS syndrome itself.^46–48^ Additionally, the *UBA1* p.Met41Val mutation may correlate with severe infections and worse outcomes.^5,46^ In our study, one deceased patient carried the p.Met41Val mutation and another had undergone prolonged corticosteroid therapy both before and after transplantation, both of whom developed opportunistic infections and subsequently died from idiopathic pneumonia syndromes.

Our multi-target real-time PCR method provides substantial practical advantages over conventional detection techniques. It is simple, cost-effective, highly sensitive, and compatible with the widely available two-color real-time PCR systems. Additionally, the short amplicon length enables the analysis of fragmented DNA from older archival samples such as paraffin-embedded tissues or bone marrow smears, as demonstrated in our skin tissue analysis, which can be challenging for traditional sequencing methods.

Despite its strengths, our method has several limitations. Accurately quantifying VAFs without digital PCR is difficult, and confirmatory analyses using either single-probe PCR or Sanger sequencing are required to identify the mutation type. Furthermore, our assay did not target minor *UBA1* variants outside the methionine-41 codon, such as splice site mutations, which account for approximately 4–7% of VEXAS syndrome cases.^8,12,13,15,49–53^ Future improvements may include multiplexing with multiple fluorochromes and additional targets or incorporating digital PCR from the initial diagnostic stage.

Another limitation is the lack of detailed clinical data on inflammatory symptoms, bone marrow vacuolation, and additional genetic testing beyond *UBA1* in Cases 1 and 2, owing to the registry-based nature of the study. Nevertheless, both patients had confirmed diagnoses of MDS, characterized by persistent cytopenia, clearly distinguishing them from clonal hematopoiesis of indeterminate potential. The diagnosis of VEXAS syndrome is based on the presence of pathogenic *UBA1* mutations*,* rather than specific clinical manifestations.^1^

In conclusion, although VEXAS syndrome is rare among unrelated HCT recipients with malignant and non-malignant BMF disorders in the historical cohort, HCT is positioned as a potentially curative, yet high-risk strategy. Additional studies are essential to refine patient selection, optimize transplant timing, and improve management strategies to mitigate risks and enhance survival. The role of tissue-residual *UBA1*-mutant clones in post-transplant complications therefore warrants further investigation.

## Supplementary Information

Below is the link to the electronic supplementary material.

Supplementary file1 (PDF 10634 kb)

## Data Availability

Further individual patient data is not publicly available due to ethical restrictions exceeding the scope of the recipient/donor's original consent for research use in the registry. Data are however available from the authors upon reasonable request and with permission of the Japanese Society of Transplantation and Cellular Therapy and the Japanese Data Center for Hematopoietic Cell Transplantation.
