# Supplementary material for: Prevalence and outcome of VEXAS syndrome in unrelated hematopoietic cell transplantation for bone marrow failure
Source: Clin Exp Med. 2025 Aug 22;25(1):300. doi: 10.1007/s10238-025-01832-7 (PMC12373688; doi:10.1007/s10238-025-01832-7)
Supplement: Supplementary file 1 — Supplementary file1 (PDF 10634 kb) [file 10238_2025_1832_MOESM1_ESM.pdf]

Supplementary Figure 1.

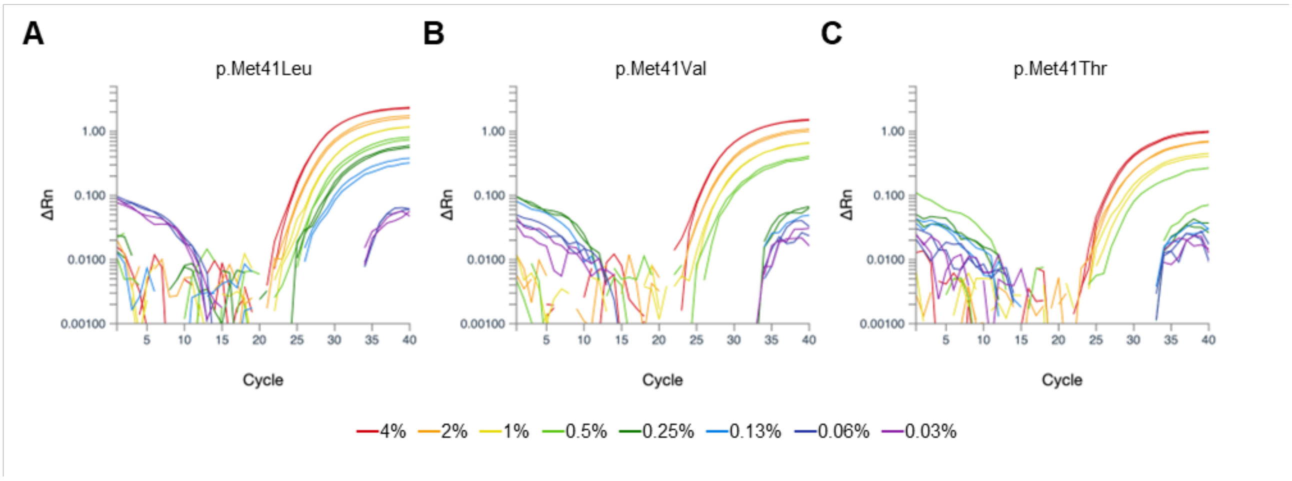

Detection limits of the multitarget real-time PCR assay. Synthesized double-stranded variant sequences were spiked into genomic DNA from a healthy male donor, and VAF was first quantified by digital PCR. In duplicate analyses using the multitarget real-time PCR assay, the detection limits were 0.13% for p.Met41Leu (**A**), 0.5% for p.Met41Val (**B**), and 0.5–1.0% for p.Met41Thr (detectable in one of two replicates at 0.5%; **C**).

Supplementary Figure 2.

A

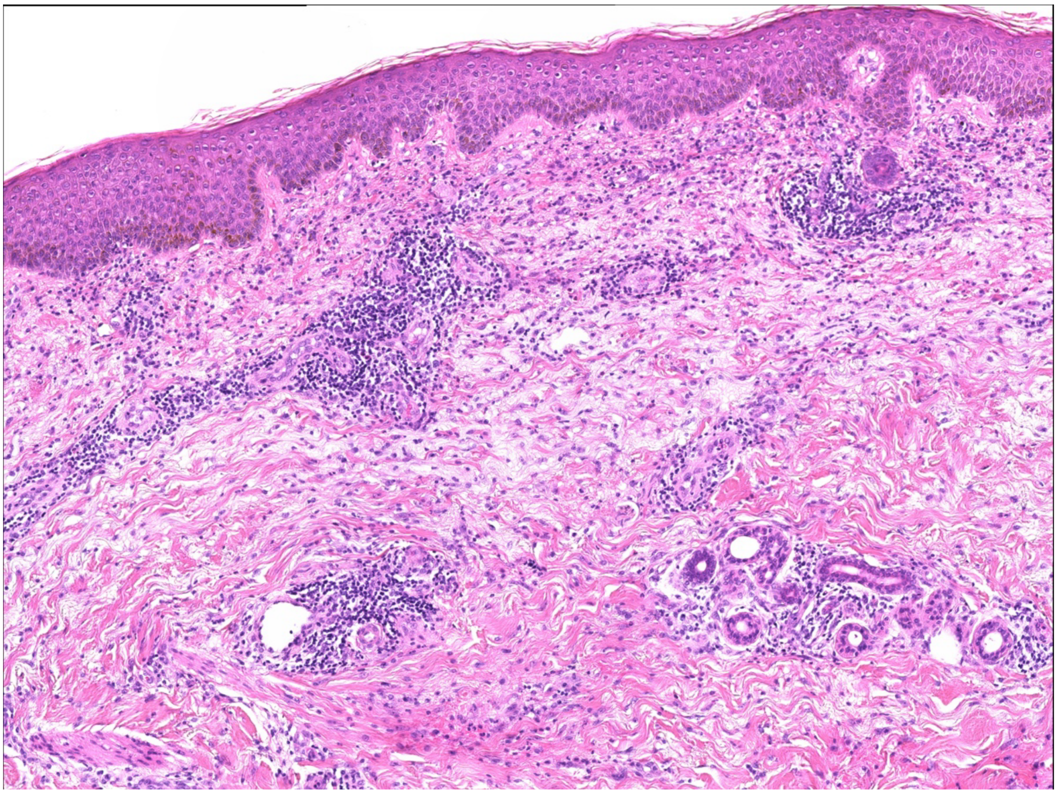

B

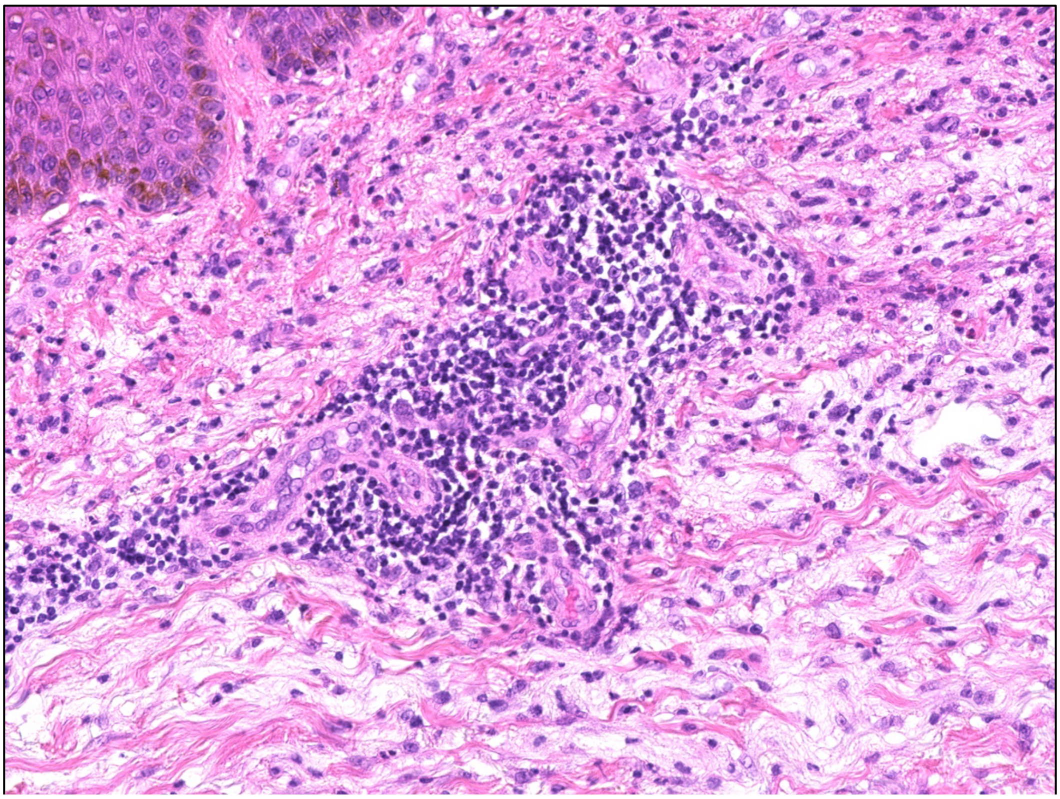

C

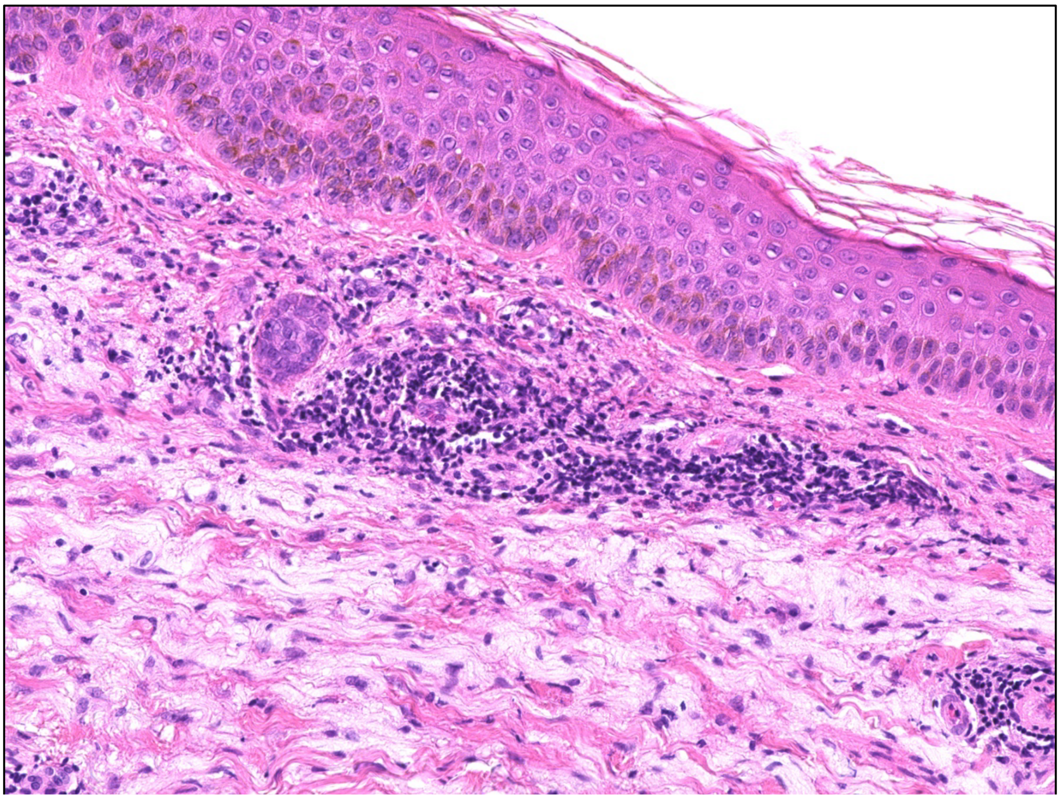

D

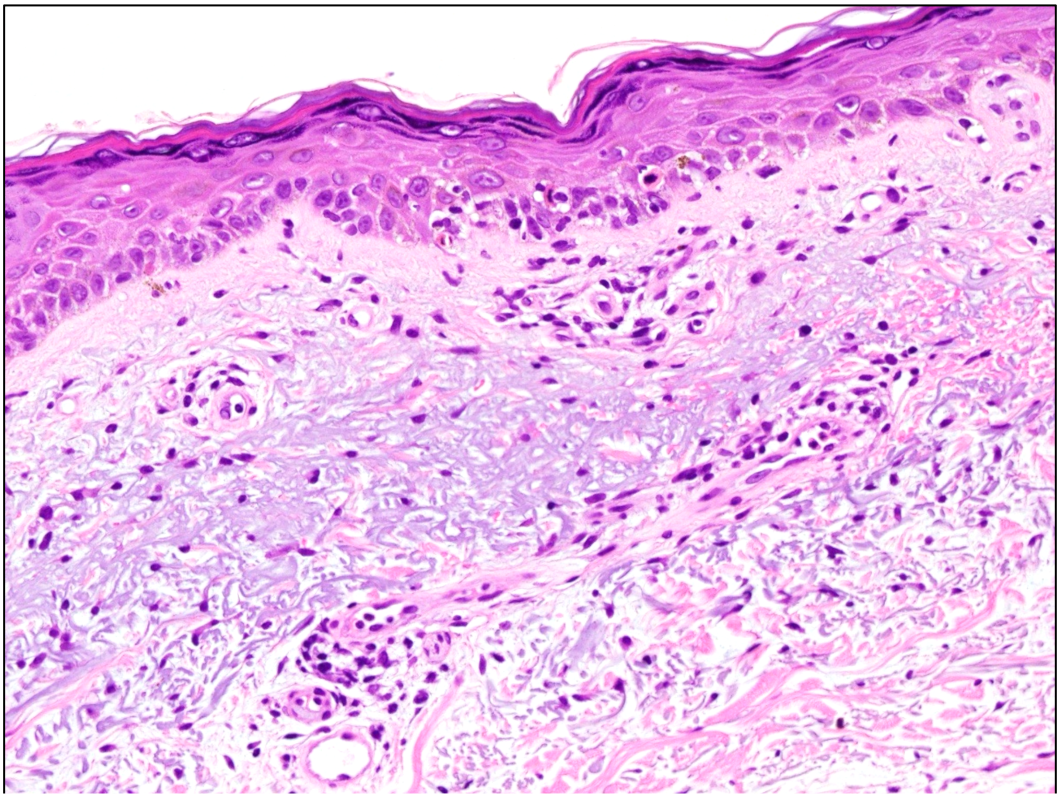

High-magnification images of hematoxylin and eosin-stained skin biopsy sections from Case 3. (A-C) Biopsy specimen obtained at the initial presentation. (D) Biopsy specimen from an acute GVHD skin lesion.

Supplementary Figure 3.

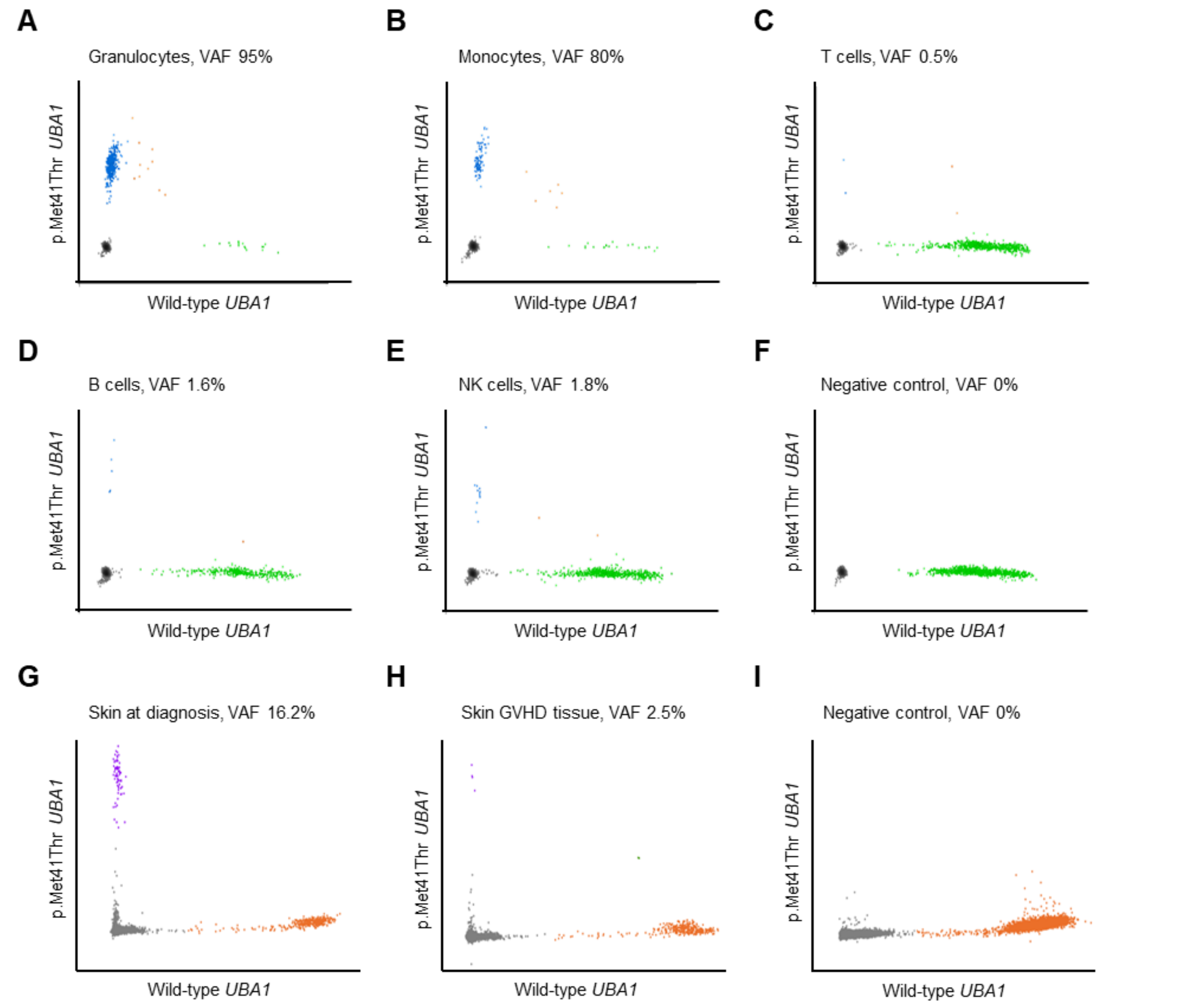

***UBA1* mutations in various cell populations.** The VAFs of the *UBA1* p.Met41Thr mutation in various cell populations from Case 3 were quantified using digital PCR. (A) $SSC^{hi}CD33^{+}$ granulocytes; (B) $FSC^{hi}CD33^{hi}$ monocytes; (C) $CD3^{+}$ T cells; (D) $CD19^{+}$ B cells; (E) $CD3^{-}$ $CD56^{+}$ NK cells; (F, I) whole blood DNA from a healthy male donor; (G) skin biopsy specimen at the diagnosis; (H) skin GVHD lesion. Samples A-F were analyzed using the QX200 Droplet Digital PCR System (Bio-Rad), and G-I were analyzed using the QuantStudio Absolute Q Digital PCR System (Thermo Fisher Scientific).

Supplementary Figure 4.

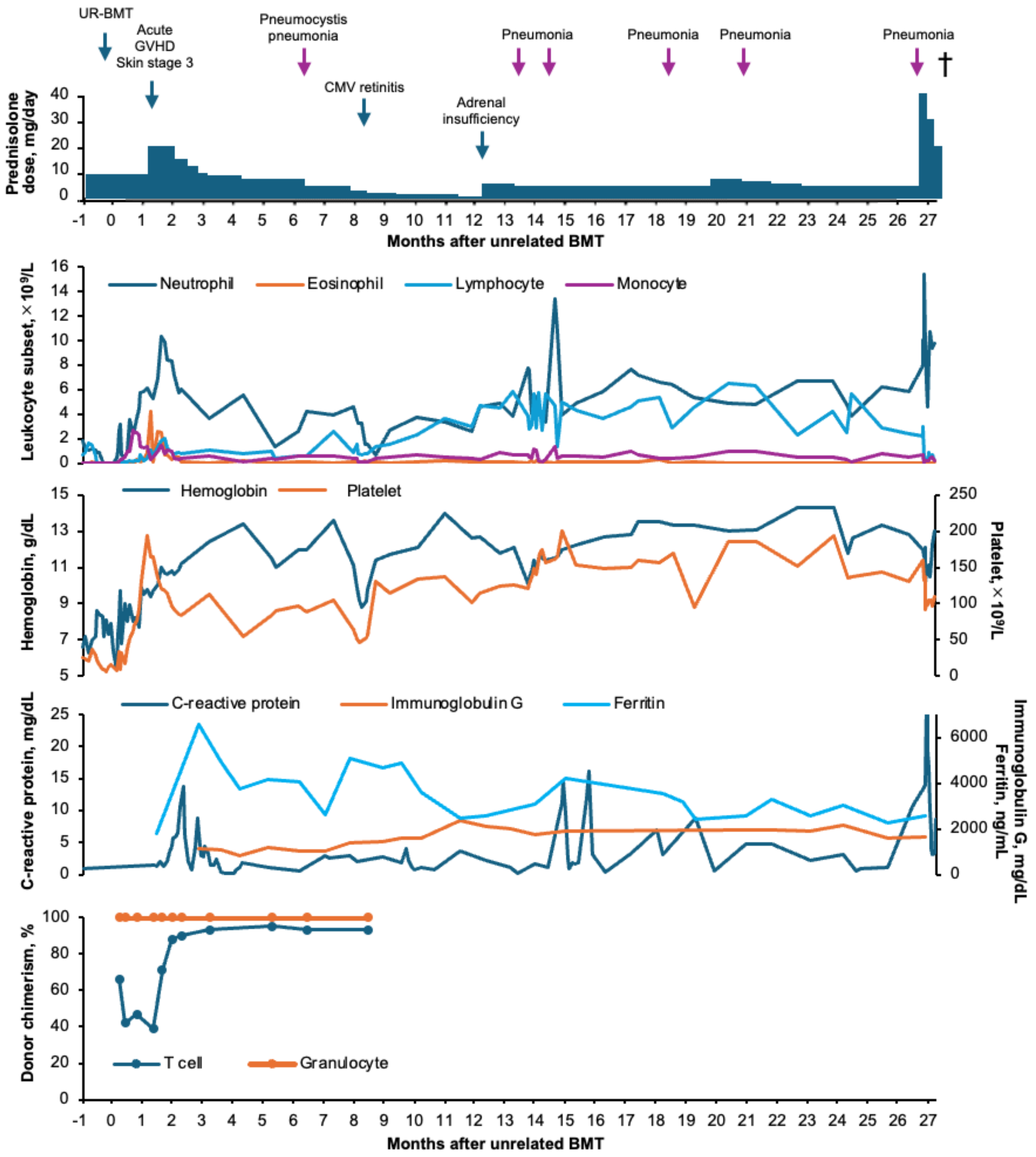

Post-transplant clinical course and immune reconstitution in Case 3.

Supplementary Figure 5.

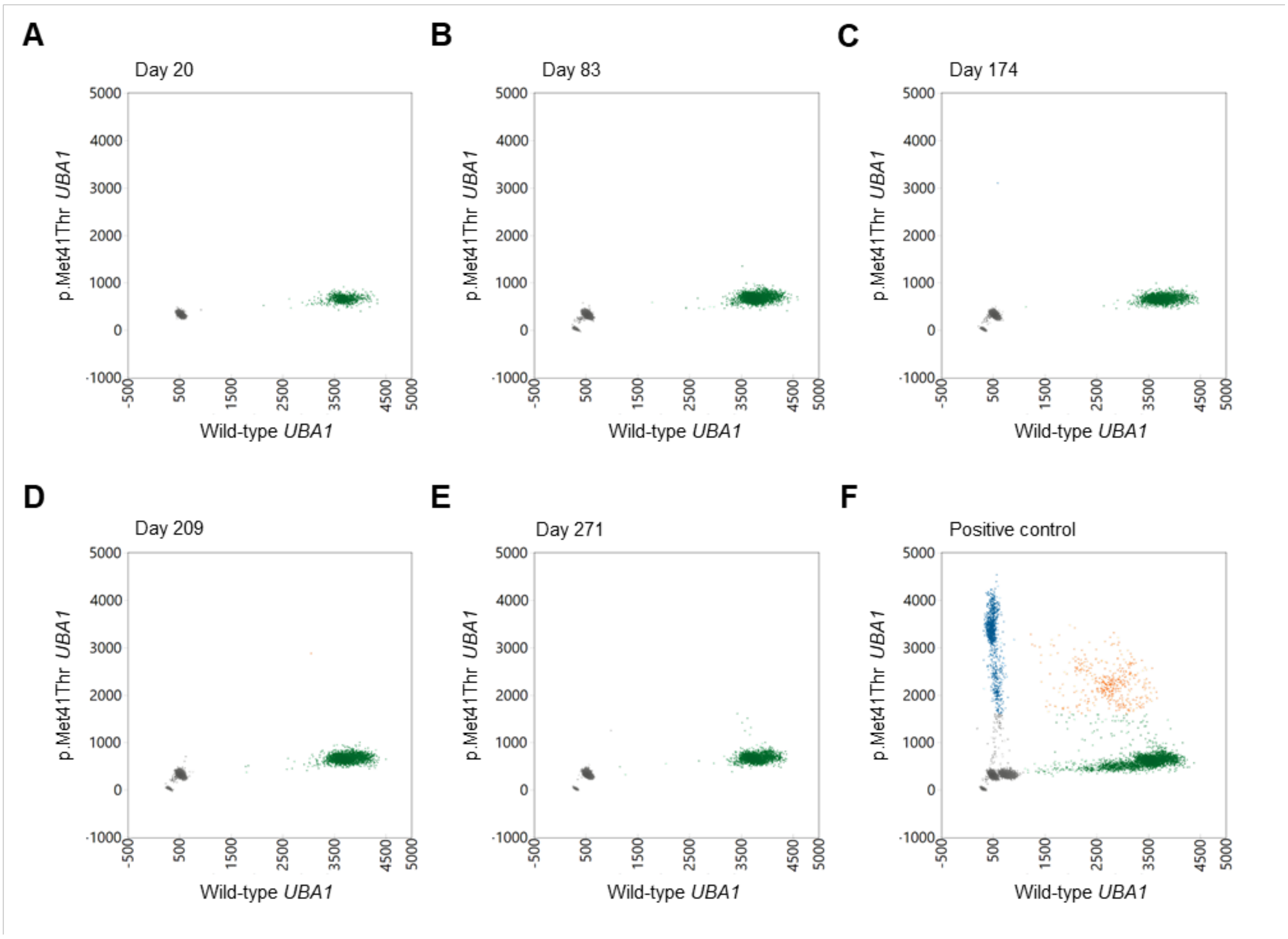

Post-transplant *UBA1* mutation analyses in Case 3. (A-E) Digital PCR analysis of post-transplant blood samples using the QX200 Droplet Digital PCR System (Bio-Rad), showing complete clearance of the *UBA1* mutation after BMT. (F) Digital PCR result of whole blood obtained before transplantation, analyzed using the same system.
